# High-throughput nuclear resonance time domain interferometry using annular slits

**DOI:** 10.1107/S1600577522002843

**Published:** 2022-04-20

**Authors:** Marc Pavlik, Dennis E. Brown, Michael Y. Hu, Jiyong Zhao, Laurence Lurio, E. Ercan Alp

**Affiliations:** a Advanced Photon Source, Argonne National Laboratory, Lemont, IL 60439, USA; b Northern Illinois University, DeKalb, IL 60115, USA

**Keywords:** nuclear resonance time domain interferometry, annular slits, relaxation times, momentum transfers, Kohlrausch–Williams–Watts model

## Abstract

Nuclear resonance time domain interferometry (NR-TDI) is used to study the slow dynamics of liquids (that do not require Mössbauer isotopes) at atomic and molecular length scales. Here the TDI method of using a stationary two-line magnetized ^57^Fe foil as a source and a stationary single-line stainless steel foil analyzer is employed. The new technique of adding an annular slit in front of a single silicon avalanche photodiode detector enables a wide range of momentum transfers (1 to 100 nm^−1^ by varying the distance between the annular slits and sample) with a high count rate of up to 160 Hz with a Δ*q* resolution of ±1.7 nm^−1^ at *q* = 14 nm^−1^. The sensitivity of this method in determining relaxation times is quantified and discussed. The Kohlrausch–Williams–Watts (KWW) model was used to extract relaxation times for glycerol. These relaxation times give insight into the dynamics of the electron density fluctuations of glycerol as a function of temperature and momentum transfers.

## Abstract 2

Nuclear resonance time domain interferometry (NR-TDI) is used to study the slow dynamics of liquids at atomic and molecular length scales. Here the TDI method of using a stationary two-line magnetized ^57^Fe foil as a source and a stationary single-line stainless steel foil analyzer is employed.

## 1. Introduction

**Figure 1:**
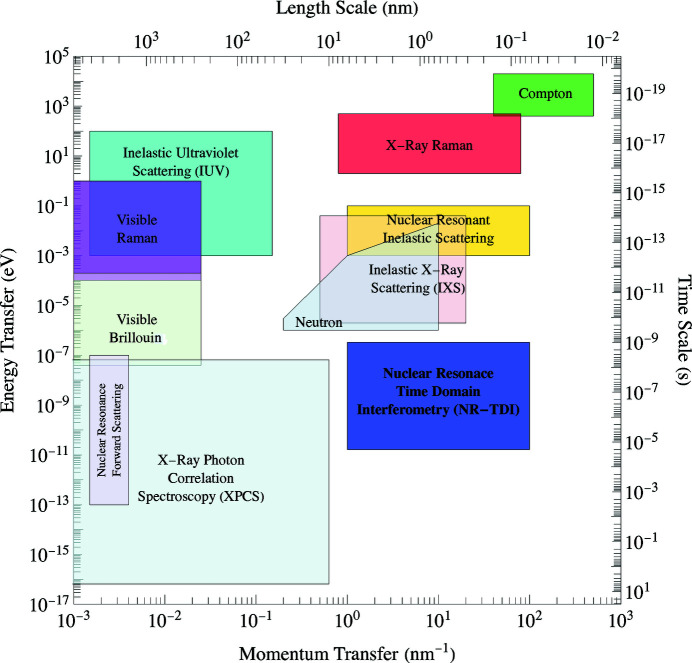
Experimental techniques that measure the dynamics of materials and the time and length scales they cover.

There are many different spectroscopy techniques covering a wide variety of time and length scales. Adding a new technique that covers a unique regime permits a deeper investigation of atomic scale dynamics for molecules, liquids, glasses, and materials in general. Fig. 1 ▸ illustrates some of the experimental techniques with the approximate time and length scales. Nuclear resonance time domain interferometry (NR-TDI) covers a range of momentum transfers between 1 and 100 nm^−1^ and energies between 0.01 and 100 neV.

In the early 1960s before the advent of TDI measurements, various Mössbauer measurements were made in an attempt to explore relaxation dynamics. Initial measurements studied the diffusion of ^57^Co or ^57^Fe ions embedded in glycerol (Craig & Sutin, 1963 ▸; Bunbury *et al.*, 1963 ▸). These early conventional transmission Mössbauer experiments studied how the motion of the ions caused diffusional broadening of the linewidths. Later, Mössbauer Rayleigh scattering experiments on ^57^Fe ions embedded in glycerol were performed (Elliott *et al.*, 1966 ▸). For these quasi-elastic scattering experiments, the diffusional broadening of the linewidths could be measured as a function of both temperature and momentum transfer. Eventually researchers realized that this scattering technique can be used to examine the motion of the individual glycerol molecules. Champeney & Woodhams (1968 ▸) used Mössbauer Rayleigh scattering to investigate the molecular motions of supercooled liquids (without embedded Fe ions) by relating how the linewidth broadening is caused by molecular diffusion and viscosity using the Stokes–Einstein equation. The dis­advantage with all these Rayleigh scattering techniques is that the counting rate is low and, thus, measurements can take up to several weeks.

The first NR-TDI experiment using synchrotron X-rays to measure the relaxation times of glycerol was performed at the European Synchrotron Radiation Facility (ESRF) (Baron *et al.*, 1997 ▸). This technique involved a moving ^57^Fe-enriched stainless steel foil (called a single line source) attached to a constant velocity drive, a liquid sample, and a stationary enriched stainless steel foil (called a single line analyzer) in front of an avalanche photodiode (APD) detector. Baron *et al.* made measurements as a function of temperature, but at only a single momentum transfer. Typical counting rates from this source/analyzer setup were around 2 Hz. This technique was improved by using a multielement detector array having eight APD detectors to measure four different scattering angles simultaneously at the ESRF beamline ID-22N (Smirnov *et al.*, 2006 ▸). This arrangement, for a particular scattering angle, allowed Smirnov *et al.* to basically increase the counting rate by a factor of two (two detectors were used to measure each angle). At the SPring-8 beamline BL09XU, this technique was modified by using a moving double line source rather than a moving single line source (Saito *et al.*, 2011 ▸). Saito *et al.* later extended his method using multiline moving sources and analyzers finding that this method increases the TDI efficiency (Saito *et al.*, 2012 ▸). Rather than using moving sources and analyzers, the technique was simplified by fixing them to be stationary. At the ESRF beamline ID18, TDI measurements were performed using a stationary double line source and single line analyzer (Caporaletti *et al.*, 2017 ▸). The stationary double line source consisted of a magnetized ^57^Fe foil, with the field perpendicular to both the direction of the beam and the incident electric field polarization. These experiments have slightly different setups but all suffer from low counting rates. We were able to improve the counting rates by a factor of two orders of magnitude using an annular slit. Using a similar setup as Caporaletti *et al.*, and placing the annular slit on the downstream single APD detector, we have been able to make practical measurements, even at low momentum transfers, which has not been achievable before.

## 2. Theory

The time domain interferometer measures the interference of electric fields in time rather than in space. One arm of our interferometer, called the source arm, consisted of a static magnetized α-^57^Fe foil, and the other arm, called the analyzer arm, consisted of a static stainless steel foil. The superposition of the electric fields generated from a two-line α-^57^Fe foil resonance and a single line stainless steel resonance gives rise to an interference spectrum in the time domain. When a sample (such as a liquid) is placed between the two arms of the interferometer, a change in the quantum beat pattern is observed. This change contains information about the relaxation dynamics of liquids. The interference spectrum, when considering only the ‘Quantum Beat’ regime, is then 

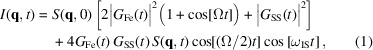

where 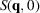 and 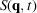 are the static and dynamic intermediate scattering functions, respectively (Smirnov *et al.*, 2006 ▸). The functions 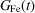 and 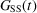 are the impulse response functions for α-^57^Fe and stainless steel resulting from an incident synchrotron X-ray pulse approximated as a delta function in time (Kagan *et al.*, 1979 ▸),

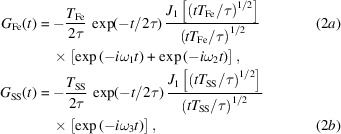

where *T* = *n*σ_0_ *fd* is the effective thickness of the iron or stainless steel foil, *n* is the number of nuclei per unit area, σ_0_ is the Mössbauer absorption cross section, *f* is the Lamb–Mössbauer factor, and *d* is the actual thickness of the foil. The lifetime of the nuclear excited state is τ = 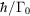 = 141.1 ns, where Γ_0_ = 4.67 neV is the natural line width of an ^57^Fe nucleus. *J*_1_ is a Bessel function of the first kind (this gives rise to dynamical beats, or ‘Bessel beats’, in the time spectra). The response function for a magnetized α-^57^Fe foil has two nuclear resonance frequencies ω_1_ and ω_2_, where Ω in equation (1) is given by Ω = ω_2_ − ω_1_. The two nuclear transition frequencies ( 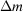 = 0) were achieved by magnetizing the iron foil with an external magnetic field perpendicular to both the incident X-ray direction 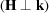 and polarization 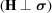 . Stainless steel is paramagnetic and was modeled to have only one resonance frequency ω_3_. The value of ω_IS_ in equation (1) is given by 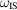 = 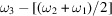 and is a slight frequency off-set due to the isomer shift of stainless steel relative to α-^57^Fe.

**Figure 2:**
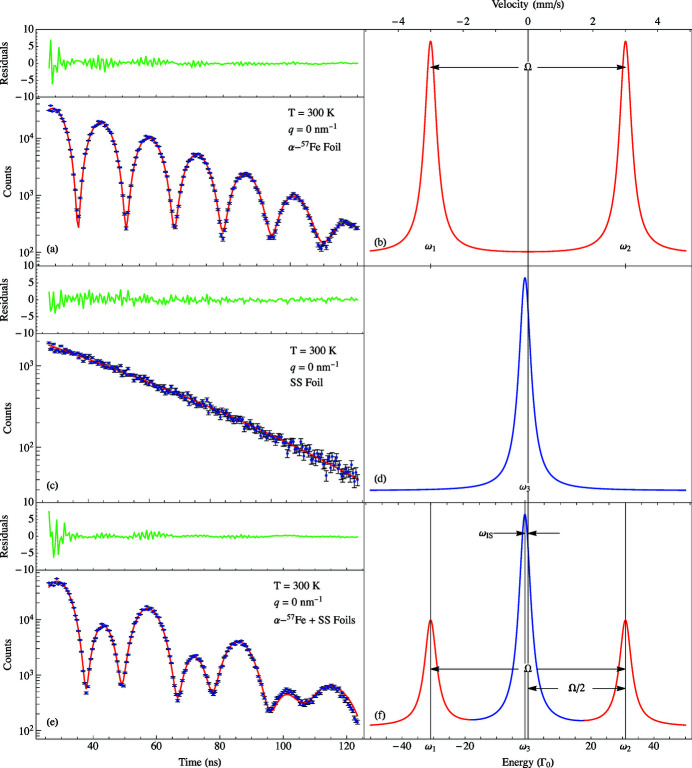
All measurements were carried out in the forward direction. (*a*) Magnetized α-^57^Fe with 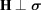 , (*b*) the two-line energy spectrum for magnetized α-^57^Fe, (*c*) stainless steel foil, (*d*) the single line energy spectrum for stainless steel, (*e*) the magnetized α-^57^Fe and stainless steel foils, (*f*) the three-line energy spectrum for the α-^57^Fe and stainless steel foils. The green lines above (*a*), (*c*), and (*e*) are the respective residuals of the fit method.

Fig. 2 ▸(*a*) shows the synchrotron Mössbauer spectroscopy (SMS) data of a magnetized α-^57^Fe foil (no sample or stainless steel foil lies in the beam). The measured intensity for such a case in the forward direction 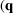 = 0) is then 

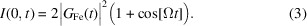

The red line in Fig. 2 ▸(*a*) is the fit with *T*_Fe_ = 15.2 ± 0.1 (corresponding to a foil thickness of 2 µm) and Ω = 0.4386 ± 0.0006 GHz (61.9 ± 0.1Γ_0_). The green line at the top in Fig. 2 ▸(*a*) describes the standardized residuals for the fit procedure, which is the difference between the fit and data divided by the standard error for each residual. Using 33 T as the standard field for an α-Fe foil, the value of Ω indicates the applied external magnetic field was 0.82 T (close to a Hall probe measurement of 0.736 T).

The measured intensity in the forward direction for an SS foil (no sample or α-^57^Fe foil lies in the beam) is 

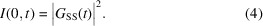

Fig. 2 ▸(*c*) shows the SMS data for the SS foil. Fig. 2 ▸(*d*) is the energy domain representation of Fig. 2 ▸(*c*). The red line in Fig. 2 ▸(*c*) is the fit with *T*_SS_ = 11.8 ± 0.1 (corresponding to a foil thickness of 0.9 µm).

For a system having only α-^57^Fe and SS foils, the scattered intensity in the forward direction is 

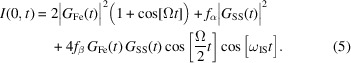

Fig. 2 ▸(*e*) shows the SMS data for the α-^57^Fe and SS foils. Fig. 2 ▸(*f*) is the energy domain representation of Fig. 2 ▸(*e*) where Ω = ω_2_ − ω_1_. The red line in Fig. 2 ▸(*e*) is the fit with ω_IS_ = −0.007 ± 0.001 GHz 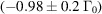 , *f*_α_ = 0.63 ± 0.04 and *f*_β_ = 0.75 ± 0.02 are factors that describe the loss of coherence due to the relative broadening of the linewidth of one absorber with respect to the other and electronic absorption.

For a particular momentum transfer and with the defined parameters discussed above (*T*_Fe_, *T*_SS_, Ω, and ω_IS_), we can determine the temperature and momentum transfer dependent relaxation times for a sample. To do so, we used the intermediate scattering function, 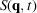 , modeled by the Kohlrausch–Williams–Watts (KWW) (Colmenero *et al.*, 1999 ▸) stretched exponential,

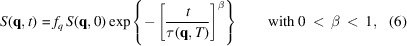

where β is the stretching coefficient, 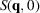 is the static intermediate scattering function and *f*_*q*_ is th non-ergodicity factor (Saito *et al.*, 2017 ▸; Caporaletti *et al.*, 2020 ▸).

Inserting equation (6) into equation (1) gives rise to the following time and momentum transfer dependent intensity after X-rays pass through the iron foil, sample, and analyzer foil where *f*_Δ*E*_ is a fitting parameter depending on the sample properties and bandwidth of the synchrotron radiation (Saito *et al.*, 2017 ▸; Caporaletti *et al.*, 2020 ▸), 

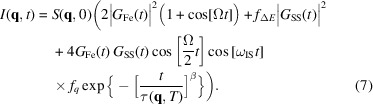

The third term in equation (7) represents the quantum beat interference between the α-^57^Fe and SS resonance lines, modified by the dynamics of a sample. The intermediate scattering function (6) of the sample suppresses this quantum beat interference. As the relaxation time τ decreases [ 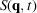 therefore decreases], this interference term gets smaller. Starting at low temperatures (long relaxation times) the quantum beat pattern initially looks similar to Fig. 2 ▸(*e*) [see Fig. 12(*b*) for glycerol]. As τ gradually decreases, the quantum beat pattern will gradually change to that of the sum of Figs. 2 ▸(*a*) and 2 ▸(*c*) which are the first two terms in equation (7) [see Fig. 12(*a*) for glycerol]. Thus, the time-dependent fluctuations in the electron density of the liquid sample will tend to destroy the coherence in the interferometer.

## 3. Experimental setup

**Figure 3:**
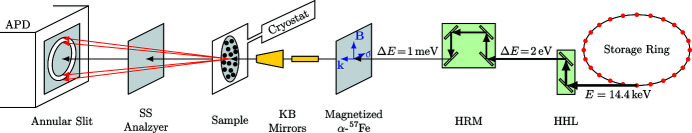
Schematic layout of the APS 3-ID beamline for NR-TDI measurements. Upstream is 2 µm magnetized (1.1 T) α-^57^Fe foil and the downstream foil is 0.9 µm ^57^Fe stainless steel (SS). The sample’s temperature is controlled with a helium flow cryostat.

The experiment was performed at Argonne National Laboratory at the Advance Photon Source (APS) at beamline 3-ID (see Fig. 3 ▸ for layout). The storage ring top-up fill pattern was 102 mA in 24 singlets having a bunch length of 33.5 ps and a bunch spacing of 153 ns. The X-ray beam was monochromated by a high-heat-load monochromator (HHL) and then by a high-resolution monochromator (HRM) to about 1 meV. The X-ray beam went through a 2 µm magnetized enriched α-^57^Fe foil with a magnetic field strength of 0.736 T which was perpendicular to both the incident X-ray direction 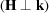 and polarization 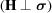 . A set of Kirkpatrick–Baez mirrors (KB) were used to focus the beam down to 15 µm onto a sample having an approximate thickness of 3 mm. The photon flux on the sample was 3 × 10^9^ photons s^−1^ meV^−1^. The sample was inside a temperature-controlled helium flow cryostat. The beam then went through a 0.9 µm enriched ^57^Fe stainless steel (SS) foil which acts like an analyzer. The 0.85 µm-thick gold annular slit makes it possible to make high-count-rate measurements at specific *q*-values. The signal was collected using a Si APD detector with an active area of 1 cm × 1 cm and a thickness of 200 µm. The detector has sub-ns time resolution and an efficiency of 40% at 14.4 keV.

**Figure 4:**
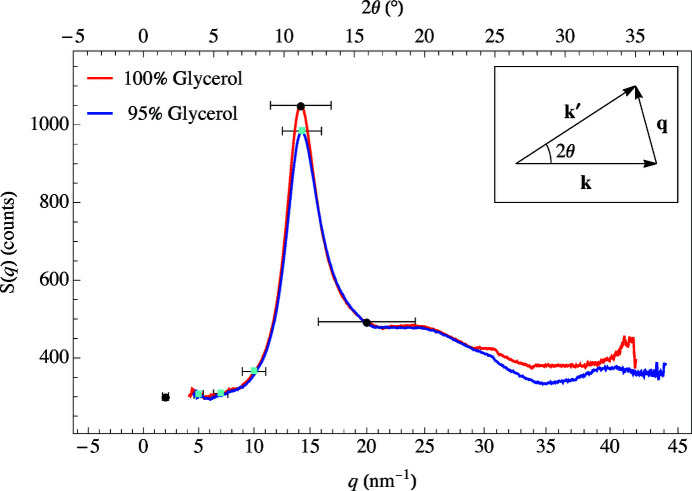
The structure factor for 100% (red line) and 95% (blue line) glycerol at room temperature. The data marks indicate where NR-TDI measurements were taken (*q* = 2, 5, 7, 10, 14, and 20 nm^−1^). The black circles are for the 1.0 mm annular slit and cyan squares are for the 0.5 mm annular slit. The error bars are the *q*-resolution of the particular momentum transfer.

A typical X-ray scattering experiment consists of an incoming wave 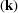 and elastically scattered wave 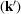 through an angle 2θ, with a momentum transfer *q* = 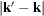 = (4π/λ)sinθ. The structure factor, *S*(*q*), is found by measuring the scattered X-ray intensity as a function of *q* or 2θ. Fig. 4 ▸ shows *S*(*q*) for 100% and 95% glycerol concentrations with the maximum scattering intensity occurring around *q* = 14 nm^−1^.

**Figure 5:**
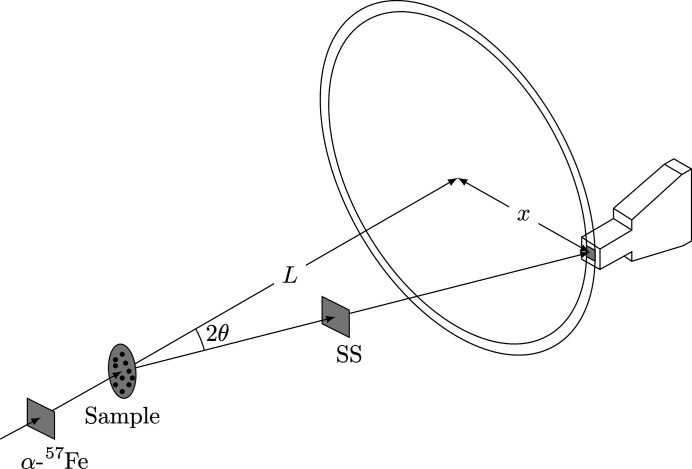
The APD has a diode area of 1 cm^2^ (shaded region). For *q* = 14.0 nm^−1^ and *L* = 75 cm, we set *x* = 14.6 cm. The area of the annulus is 91.7 cm^2^.

An early approach at NR-TDI measurements used two stationary foils as shown in the setup in Fig. 5 ▸, where the distance between the sample and APD detector is *L*, and *x* is the transverse distance of the APD detector.

The momentum transfer, *q*, is defined by 

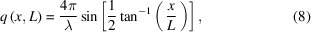

where λ = 0.086 nm. To get *q* = 14.0 nm^−1^ for *L* = 75 cm, we had to set *x* = 14.6 cm. For this geometry, X-rays will be scattered into an annulus of area 91.7 cm^2^ for an APD detector having an active diode area of 1 cm × 1 cm. Using this geometry we are capturing approximately 1% of the total X-rays scattered into the annulus (shaded region in Fig. 5 ▸).

Some previous experiments have used multiple detectors and at multiple angles to combat the inherent low counting rate (Caporaletti *et al.*, 2017 ▸; Saito *et al.*, 2011 ▸). However, it takes specialized equipment and electronics. Even then only a small portion of the X-rays scattered into the annulus shown in Fig. 5 ▸ are collected.

**Figure 6:**
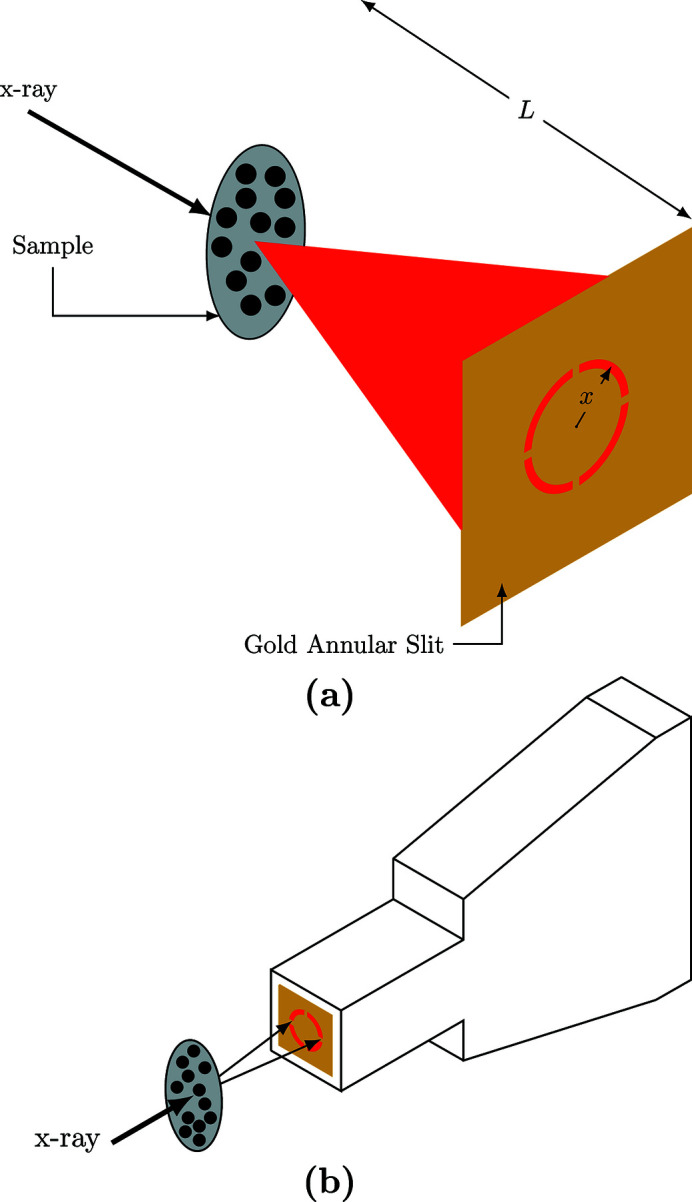
Scattering setup for the gold annulus, where *L* is the distance from the sample to the annulus and *x* is the radius to the center of the slit. For the 1.0 mm-wide annular slit the radius is *x* = 4.0 mm. To measure *q* = 14.0 ± 2.7 nm^−1^, *L* must be 20.6 mm.

In an attempt to collect all the X-rays scattered at a particular *q*, we built an annular slit which can be positioned in front of an APD detector, as shown in Fig. 6 ▸. This way we were able to collect 92% of the scattered X-rays instead of the 1% from the single detector setup shown in Fig. 5 ▸. This resulted in an increase in count rate of almost two orders of magnitude.

**Figure 7:**
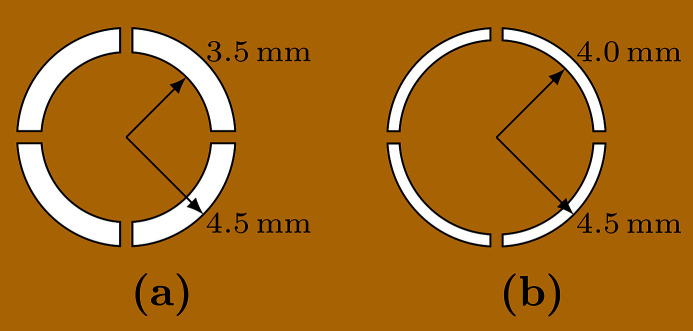
(*a*) 1.0 mm and (*b*) 0.5 mm aperture annular slits with a thickness of 85 µm used to measure *q*.

Two annular slits with 1.0 mm and 0.5 mm apertures were made using a wire EDM machine. This machine uses a wire electrode to precisely cut contours into a workpiece. The annular slits were made from a 20 mm × 20 mm-wide 85 µm-thick gold foil. An extra piece of gold was added to the center of the annular slit in order to prevent the direct beam from entering into the APD detector. The dimensions of the annular slits are shown in Fig. 7 ▸. A custom mount was designed that attached to the APD allowing the annular slits to be precisely centered and placed very close to the detector.

**Figure 8:**
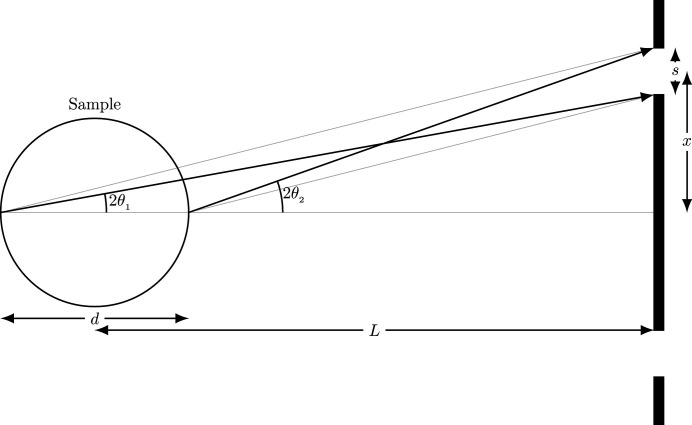
Diagram showing the angles used to calculate the resolution in *q*, Δ*q*.

**Figure 9:**
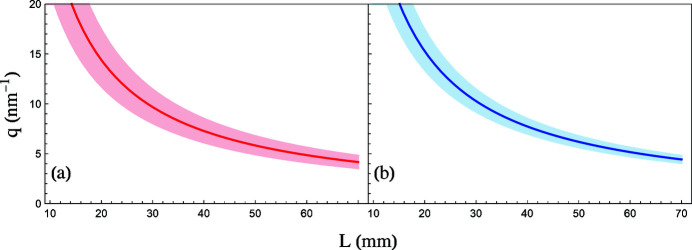
The dependence of *q* and Δ*q* as a function of distance. The solid line represents *q*-values from equation (9) assuming a sample width of 3 mm. The shaded region represents Δ*q* accounting for the aperture sizes of (*a*) 1.0 mm and (*b*) 0.5 mm. As can be seen in the two figures above, Δ*q* becomes larger at large momentum transfer values, which can limit on how high a momentum transfer is measurable; in that case one can resort to even smaller annular slit widths albeit at reduced data collection rate.

The scattered X-ray intensity at the detector depends on three factors: (1) the distance from the sample *L*, (2) the thickness of the sample *d*, and (3) the width of the slit *s*. This is shown schematically in Fig. 8 ▸. From equation (8), the angle for finite *q* is defined by

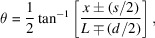

where *x* is the distance from the center of the annulus to the center of the slit. For the two extreme angles shown in Fig. 8 ▸, where θ_2_ > θ_1_, the spread in *q* is 

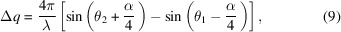

where α is the angular spread of the incident beam. When *L* ≫ *d*, the sample thickness can be ignored and, as *L* increases, the difference between θ_2_ and θ_1_ becomes smaller, thereby reducing Δ*q*. Similarly, reducing the aperture size also decreases Δ*q*; however, the downside is a decrease in the counting rate. At larger *q* values the thickness of the sample plays a major role in the Δ*q* resolution, whereby doubling the thickness nearly doubles the Δ*q* resolution. Fig. 9 ▸ shows how increasing *L* decreases Δ*q* for the two different aperture sizes.

When choosing the size of the slit, there is a trade-off between the *q*-resolution and counting rate. A large slit increases the counting rate at the cost of *q*-resolution, and a small slit increases the resolution at the expense of lower count rates. We used two different slit widths (1 mm and 0.5 mm) having Δ*q* ≃ 5 nm^−1^ and Δ*q* ≃ 3.5 nm^−1^, respectively, at *q* = 14 nm^−1^. As shown in Table 1 ▸, it is clear that there is a trade-off between count rate and *q*-resolution. With the annular slits we managed to increase the count rate by about two orders of magnitude. This, in turn, enabled us to make measurements at *q*-values previously unexplored.

## 4. Results

TDI measurements are notoriously difficult to perform using either radioactive sources or synchrotron X-rays due to low count rates. This is because the intensity of the quasielastic scattered photons is five to six orders of magnitude lower than the direct beam. To overcome this problem we developed a new optical component: an annular slit. The annular slit allows for the collection of all scattered photons for a particular momentum transfer using only one APD detector. This improvement, in turn, allows systematic studies of relaxation times over a range of momentum transfers and temperatures, and it presents an advantage over previous approaches (Baron *et al.*, 1997 ▸; Saito *et al.*, 2017 ▸; Caporaletti *et al.*, 2017 ▸).

Attaching an annular slit to an APD detector, as shown in Fig. 6 ▸(*b*), allows for a much higher counting rate and detection efficiency. For instance, using a detector with a 1.0 mm annular slit [Fig. 7 ▸(*a*)] has an average delayed counting rate of 90 Hz at *q* = 14 nm^−1^ and *T* = 300 K for glycerol. Using the single detector setup shown in Fig. 5 ▸, we measured an average delayed counting rate of 0.5 Hz for the same momentum transfer and temperature. However, the annular slit has a *q*-resolution of 5 nm^−1^ compared with 1 nm^−1^ for the single detector setup shown in Fig. 5 ▸ (since the single detector was placed much further from the glycerol sample). With annular slits, it was possible to collect data at low *q*-values where the scattering intensity is naturally lower than the peak intensity of *S*(*q*) (Fig. 4 ▸). Table 1 ▸ lists the counting rates and relaxation times for multiple *q*-values and slit sizes at *T* = 300 K for the 100% glycerol sample. These counting rates make it possible to carry out measurements at momentum transfers as low as 1.9 nm^−1^.

**Figure 10:**
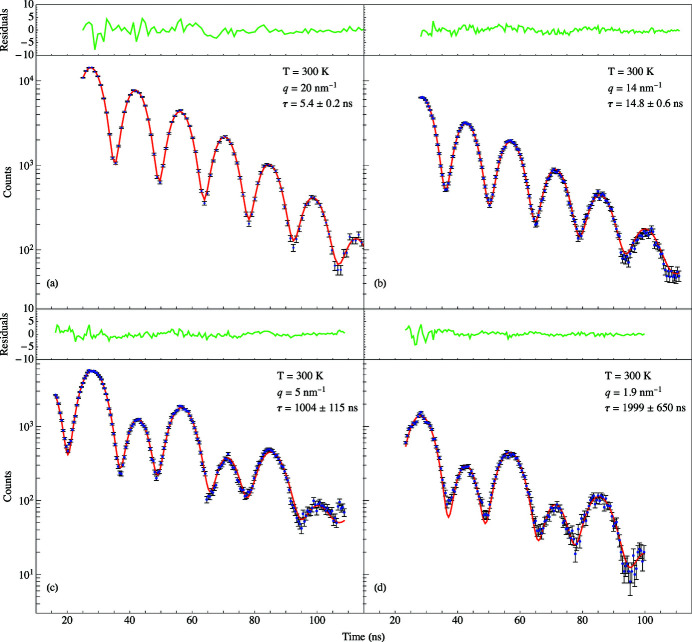
TDI results for 100% glycerol at 300 K at four different momentum transfers (*a*) *q* = 20 nm^−1^, (*b*) *q* = 14 nm^−1^, (*c*) *q* = 5 nm^−1^, and (*d*) *q* = 1.9 nm^−1^. Blue dots: experimental data. Red solid line: fit obtained using equation (8). The green lines above the plots are the respective residuals for the fits below.

**Figure 11:**
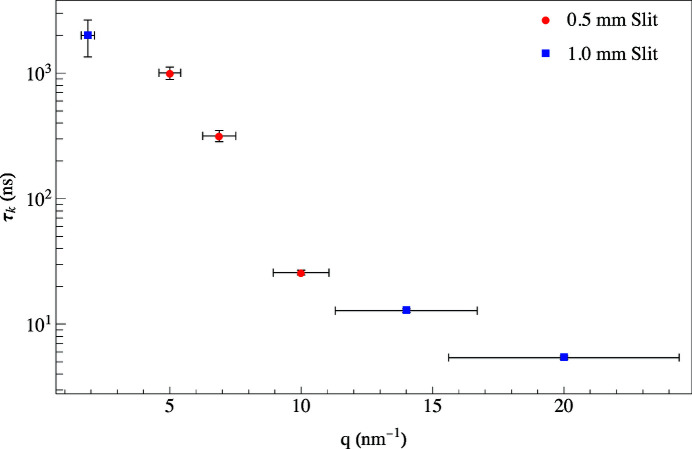
Calculated relaxation times using two different annular slits for 100% glycerol at *q* = 2, 5, 7, 10, 15, and 20 nm^−1^ at 300 K. The blue squares are for the 1.0 mm and the red circles are for the 0.5 mm slit widths.

The dynamic intermediate scattering function given by equation (6) determines the degree of coherence as measured by the time domain interferometer. The KWW stretched exponential function contains information about fluctuations in the electron density of the sample due to the dynamic diffusive motion of its molecules. The relaxation time 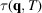 is found by fitting the quantum beat spectra to equation (7). Notice the dramatic change in the time spectra as *q* is varied in Fig. 10 ▸. At *T* = 300 K when *q* was set to 14 nm^−1^, a spectrum was measured having a relaxation time of 14.8 ± 0.6 ns. For such a short relaxation time, the KWW function is small which in turn makes the coherence term in equation (7) (the third term) small. Thus, the interference between the Fe and SS foils has been greatly reduced resulting in the spectrum shown in Fig. 10 ▸(*a*). At smaller *q* values, the KWW function becomes larger which in turn makes the coherence term in equation (7) larger. This results in the significantly modulated spectrum shown in Fig. 10 ▸(*b*) for *q* = 1.9 nm^−1^ for the same temperature. This spectrum has a much longer relaxation time of 1999 ± 650 ns indicative of a reduction in the fluctuating, diffusive motion of the glycerol molecules and thus having little effect on the interference between the Fe and SS foils. Fig. 11 ▸ shows a plot of relaxation times as a function of momentum transfers for the two different aperture sizes. The *q*-resolution improves at lower momentum transfers because the detector had to be placed further from the sample in order for the X-rays to make it through the annular slit. The benefit of using the wider aperture is a higher counting rate, but there is a sacrifice in the *q*-resolution because the detector must be placed closer to the sample.

**Figure 12:**
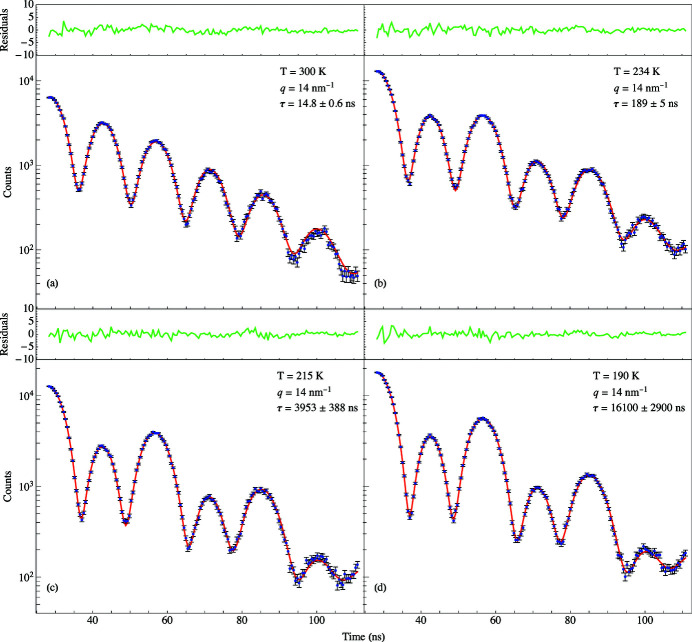
TDI results for 100% glycerol at *q* = 14 nm^−1^ and four different temperatures (*a*) *T* = 300 K, (*b*) *T* = 234 K, (*c*) *T* = 215 K, and (*d*) *T* = 160 K. Blue dots: experimental data. Red solid line: fit obtained using equation (8). The green lines above the plots are the respective residuals for the fits below.

The relaxation time increases when *q* decreases thereby enhancing the interference between the Fe and SS foils. The relaxation time can also be increased by decreasing the temperature at constant *q*. This can be seen in the significantly modulated spectrum shown in Fig. 12 ▸(*b*) for the low temperature of 190 K having a relaxation time of 16100 ± 2900 ns. For such a long relaxation time the diffusive motion of the glycerol molecules is almost static. At the higher temperature of 300 K shown in Fig. 12 ▸(*a*), the relaxation time is much smaller (14.8 ± 0.6 ns) and the interference between the foils is reduced resulting in very little modulation of the spectrum.

**Figure 13:**
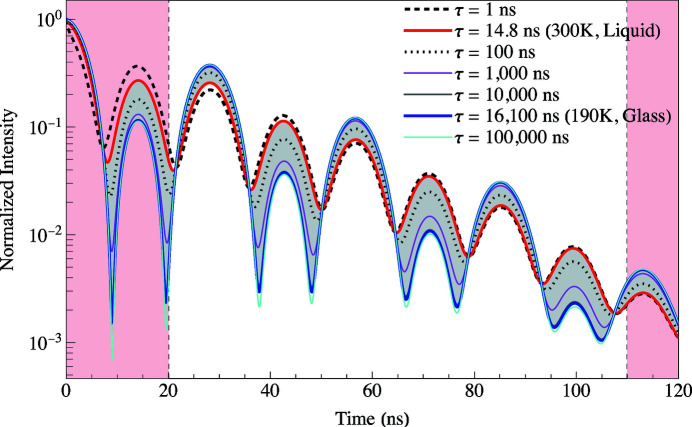
Normalized intensities for various relaxation times. The gray shaded region spans the measurements made in Table 2 ▸. The curves outside the gray shaded region are theoretical calculations for relaxation times and the measurement window is between the red shaded regions (20 to 110 ns).

The sensitivity of the NR-TDI technique to measuring the relaxation time can be determined by the degree of modulation of the time spectra. This is shown in Fig. 13 ▸ where the gray shaded modulation region shows how the time spectrum evolves from a short relaxation time (solid red curve) to the heavily modulated spectrum having a long relaxation time (solid blue curve). The calculations are all normalized to unity at time *t* = 0 ns. The gray shaded region covers the series of measurements given in Table 2 ▸ for the 1 mm annular slit. From the degree of modulation of the spectra, it appears to be possible to measure relaxation times over a large dynamic range covering four orders of magnitude from 1 ns to at least 10000 ns (as shown in Fig. 1 ▸).

**Figure 14:**
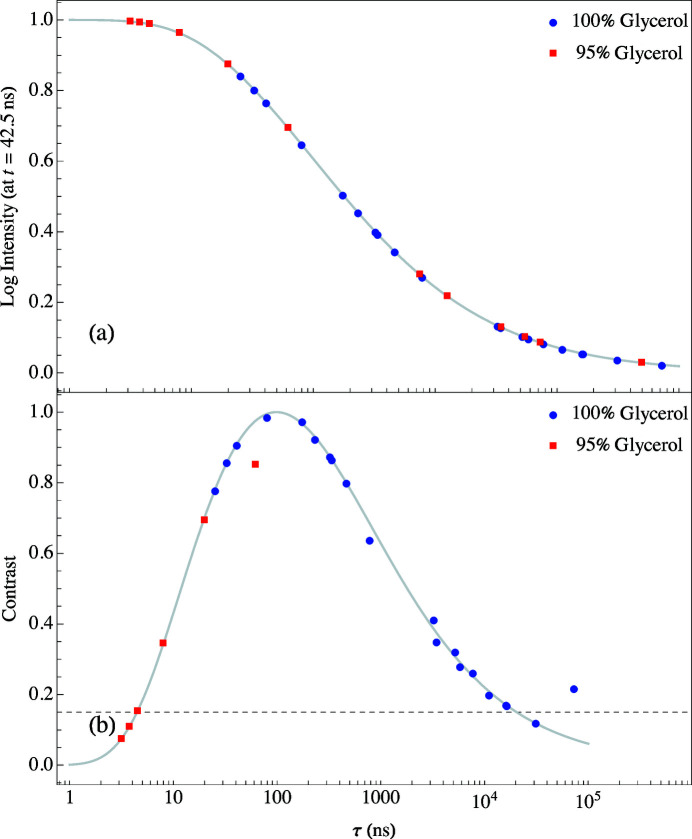
Sensitivity curves for NR-TDI measurements using 100% glycerol where the smooth curves are simulations and the solid circles are evaluations at the values of τ given in Table 2 ▸. (*a*) Log intensity at *t* = 42.5 ns in Fig. 13 ▸, (*b*) the contrast is the derivative of the curve in (*a*).

The log of the intensity, at *t* = 42.5 ns in Fig. 13 ▸, as a function of relaxation time is the red curve shown in Fig. 14 ▸(*a*). The log of the intensity curve was adjusted so that it is 1 at *t* = 0 and 0 at *t* = ∞. The solid black circles are from the 26 measurements given in Table 2 ▸ for 100% glycerol and the solid magenta circles are from the 14 measurements given in Table 2 ▸ for 95% glycerol. The curve plateaus for times less than 1 ns and greater than 100000 ns showing that experiments are not very sensitive to measuring relaxation times in those regions. The normalized contrast curve in Fig. 14 ▸(*b*) is the derivative of the red curve, d(log Intensity)/d(log τau), showing that the peak sensitivity for this particular NR-TDI technique lies around 100 ns. When the error bars of the relaxation times are greater than 10%, the contrast value falls below 15%, indicating the limitation of reliable measurements. For a 15% contrast the sensitivity range from 5 ns to 20000 ns is shown by the dashed line in Fig. 14 ▸(*b*).

Glycerol is a well studied material that has relaxation times in the range we can measure (1 to 7000 ns). The relaxation times were determined by fitting the data to equation (7) using a Levenberg–Marquardt least-squares fit where the uncertainties were given by the covariance matrix. Using the 1.0 mm annular slit for 100% glycerol resulted in an average delayed count rate of 40 Hz, whereas the 0.5 mm annular slit measurement for 95% glycerol had an average delayed count rate of 15 Hz. The 95% by volume concentration of glycerol to 5% deionized ultra-filtered water has a slightly lower *S*(*q*) peak at 14 nm^−1^ (Fig. 4 ▸).

**Figure 15:**
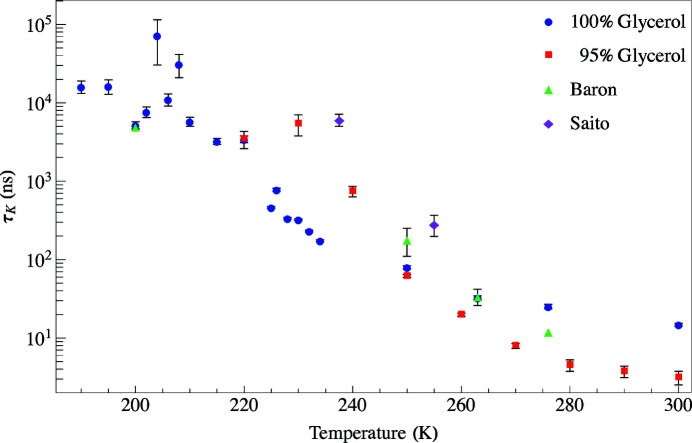
Relaxation times as a function of temperature for 100% (blue circles using 1.0 mm annular slit) and 95% (red squares using 0.5 mm annular slit) glycerol at the peak of the structure factor, *q* = 14 nm^−1^. Included in the plot are fits for glycerol from Baron *et al.* (1997 ▸) and Saito *et al.* (2017 ▸).

Plots of the relaxation times as a function of temperature for both concentrations are shown in Fig. 15 ▸. For the glycerol samples there is a noticeable glass transition temperature around 195 K which agrees reasonably well with that reported in the literature (*T*_g_ = 190 K). At higher temperatures we can observe relaxation dynamics well before the melting point reported in the literature (*T*_m_ = 291 K). There is a significant increase in the diffusive motion of glycerol molecules over a 40 K temperature span before the onset of melting where the material is in a soft glassy phase (Zondervan *et al.*, 2007 ▸, 2008 ▸; Möbius *et al.*, 2010 ▸). We found the discussion by Caporaletti *et al.* (2017 ▸) quite illuminating in extraction of relaxation time by TDI.

## 5. Conclusion

We introduce a new optical component, a gold annular slit, that increases the count rate by two orders of magnitude in NR-TDI measurements. Measurements at different *q*-values [as well as *S*(*q*) measurements] can be made by simply varying the sample-to-slit distance. We present a clear formula representing the response of a stationary resonant three-line two-foil system and a non-resonant liquid sample. We carried out extensive numerical analysis to determine the contrast and sensitivity of the experiment. For glycerol we performed temperature-dependent measurements over a wide range of momentum transfers ranging from 2 to 20 nm^−1^, improving the understanding of the molecular dynamics of glycerol.

Due to the large increase in counting rate, it is now possible to perform measurements at low momentum transfers that were once considered unrealistic. We made measurements at momentum transfers of *q* = 2, 5, 7, 10, 14 and 20 nm^−1^ and at temperatures ranging from 190 K to 300 K using this technique. The annular slits allows for the collection of almost the entire ring of scattered X-rays at any *q* between 1 nm^−1^ and 100 nm^−1^ and relaxation times between 1 ns and 10000 ns using a single APD detector.

## Figures and Tables

**Table 1:** Counting rates and relaxation times for multiple *q*-values and slit sizes at *T* = 300 K for 100% glycerol The mean relaxation time can be calculated by the formula 〈τ〉 = (τ/β)Γ(1/β) ≃ 1.5τ (β was fixed to 0.6 for all fits).

Slit size (mm)	Momentum transfer (nm^−1^)	Count rate (Hz)	τ (ns)
1.0	1.9 ± 0.3	1.76	1999 ± 650
0.5	5.0 ± 0.4	8.19	1004 ± 115
0.5	6.9 ± 0.6	14.7	317 ± 32
0.5	10.0 ± 1.1	53.2	25.8 ± 1.2
1.0	14.0 ± 2.7	106.6	14.8 ± 0.6
1.0	20 ± 4	13.9	5.4 ± 0.2

**Table 2:** Table of relaxation times at multiple temperatures for 100% and 95% glycerol using 1.0 mm and 0.5 mm annular slits at 14 nm^−1^ corresponding to the average inter-molecular distance of glycerol The mean relaxation time can be calculated by the formula 〈τ〉 = (τ/β)Γ(1/β) ≃ 1.5τ (β was fixed to 0.6 for all fits).

	100% glycerol	95% glycerol
	1.0 mm annular slit	0.5 mm annular slit
*T* (K)	τ (ns)	τ (ns)
190	16100 ± 2900	
195	16300 ± 3500	
200	5186 ± 556	
202	7700 ± 1200	
204	70 000 ± 40 000	
206	11000 ± 2000	
208	31000 ± 10000	
210	5803 ± 775	
215	3234 ± 296	
220	3437 ± 338	3456 ± 845
225	463 ± 12	
226	780 ± 33	
228	336 ± 9	
230	323 ± 7	5400 ± 1600
232	232 ± 6	
234	174 ± 4	
240		743 ± 115
250	80 ± 4	61.8 ± 2.9
260		19.9 ± 1
263	32.7 ± 1.9	
270		7.9 ± 0.6
276	25.2 ± 1.7	
280		4.5 ± 0.7
290		3.7 ± 0.6
300	14.8 ± 0.6	3.1 ± 0.6
